# Genome Sequence of the Pigment-Producing Bacterium *Pseudogulbenkiania ferrooxidans*, Isolated from Loktak Lake

**DOI:** 10.1128/genomeA.01115-13

**Published:** 2013-12-26

**Authors:** Sampada Puranik, Reshma Talkal, Asifa Qureshi, Anshuman Khardenavis, Atya Kapley, Hemant J. Purohit

**Affiliations:** Environmental Genomics Division, National Environmental Engineering Research Institute (CSIR-NEERI), Nehru Marg, Nagpur, India

## Abstract

The whole genome of a pigment-producing isolate from a lake in northern India, *Pseudogulbenkiania ferrooxidans* strain EGD-HP2, has been sequenced to study the spectrum of biosynthesis of secondary metabolites. The genome annotation data revealed an operon for violacein, which showed homology with the reported operon of a *Chromobacterium* sp., and also a quinone cofactor.

## GENOME ANNOUNCEMENT

A pigment-producing strain was isolated from Loktak Lake in northern India. To understand the possible nature of the pigment, whole-genome sequencing of the isolate was done. The draft genome data were analyzed for biosynthesis of pigment and secondary metabolites. According to the annotated data, the genome was confirmed to be that of a *Pseudogulbenkiania ferrooxidans* strain. The functional categories of genes were identified through the SEED Viewer. Of the various secondary metabolic pathways, we identified the complete operon for violacein biosynthesis. The operon consisted of all the genes which are involved in biosynthesis of violacein, including *vioA* (tryptophan-2-monoxygenase), *vioB* (violacein biosynthesis protein), *vioC* (monoxygenase), *vioD* (tryptophan hydroxylase), and *vioE* [proto(deoxy)violaceinic acid synthase], along with important regulatory elements (1, 2). The violacein pathway of this strain shows homology with the reported pathway from *Chromobacterium violaceum*. An additional interesting feature of this strain, which we mapped using RAST (Rapid Annotations using Subsystems Technology) (3, 4), is the presence of a dehydrogenase flavoprotein, a quinone cofactor, encoded by the *lodB* gene.

The pathway analysis shows the presence of various important subcategories, like secondary metabolism, stress response (mainly to oxidative stress), iron acquisition and metabolism, and aromatic compound metabolism. These are the subcategories involved in distant cause-effect relationships, with the violacein production being under the influence of a quorum-sensing mechanism. Identification of such genes and investigations of the details of promoter regulation through sequence analysis using B-PROM could reveal strategic interrelations of networking, which might elucidate the molecular aspects of dye production (5, 6). These data might open new avenues into industrial production of secondary metabolites. Natural pigments, like violacein, also display advantageous biological activities, such as antiprotozoal, antileishmanial, antioxidant, antibacterial, and anticancer effects (7–10). With sequencing technology becoming more affordable, the draft-genome approach could be used for rapid screening of pigment-producing strains from unique habitats with potential secondary metabolites of commercial value.

The high-quality reads of 387,863 bp were assembled into 301 contigs by use of GS Assembler/CLC Genomics Workbench with optimized parameters (v2.3). The draft genome sequence has an average GC content of 64.1%. The genome was annotated using RAST v4.0 and the NCBI Prokaryotic Genomes Automatic Annotation Pipeline (PGAAP) (http://www.ncbi.nlm.nih.gov/genomes/static/Pipeline.html). NCBI PGAAP annotated 301 high-quality contigs into genes, 4,392 coding sequences (CDS), 80 tRNA genes, 4 copies of 5S rRNA genes, 1 copy of a 16S rRNA gene, and 8 pseudogenes.

### Nucleotide sequence accession number.

This whole-genome shotgun project has been deposited at DDBJ/EMBL/GenBank under the accession no. AVPH00000000. The version described in this paper is the first version.
